# Salivary macrophage chemokines as potential biomarkers of gingivitis

**DOI:** 10.1186/s12903-023-02787-5

**Published:** 2023-02-06

**Authors:** Amna Alhammadi, Aghila Rani Koippallil Gopalakrishnan, Roba Saqan, Zahi Badran, Sausan Al Kawas, Betul Rahman

**Affiliations:** 1 Master of Dental Surgery in Periodontics, College of Dental Medicine, University of Sharjah, Sharjah, UAE; 2 Wound Healing and Oral Diagnostic Research Group-Sharjah Institute of Medical Research, University of Sharjah, Sharjah, UAE; 3 Research Institute for Medical and Health Sciences, University of Sharjah, Sharjah, UAE; 4 Department of Preventive and Restorative Dentistry, College of Dental Medicine, University of Sharjah, Sharjah, UAE; 5 Department of Oral and Craniofacial Health Science, College of Dental Medicine, University of Sharjah, Sharjah, UAE

**Keywords:** Saliva, Gingivitis, Biomarkers, MAF, MIF, MCF

## Abstract

**Objective:**

The present study aimed to analyze the salivary levels of macrophage-activating factor (MAF), macrophage-chemotactic factor (MCF), and macrophage migration inhibitory factor (MIF) in healthy and gingivitis patients, and to correlate between the concentrations of these chemo attractants with the intensity of gingival inflammation clinically.

**Methods:**

Sixty saliva specimens were collected from periodontally healthy (n = 30), and gingivitis patients (n = 30). Bleeding on probing (BOP), Visible Plaque Index (VPI), and Simplified Modified Gingival Index (SMGI) were recorded through clinical examination. Salivary MAF, MCF, and MIF concentrations were assayed using enzyme-linked immunosorbent assays (ELISA). Statistical analysis was performed using SPSS (version 28). Total mean score for each biomarker was determined, and descriptive bivariate statistics were conducted to characterize the levels of biomarkers among the study groups. The difference in the biomarker levels among the study groups were analyzed by independent sample t test and one-way ANOVA. The diagnostic ability of the biomarkers was further tested by ROC curve analysis.

**Results:**

Salivary levels of MAF was not significantly different between periodontally healthy individuals and gingivitis patients. The difference in MCF and MIF levels between patients with gingivitis and those with healthy periodontium was statistically significant (*p* 0.05 and *p* 0.001, respectively). When examined across the various stages of disease progression, MIF showed statistically significant difference among the three biomarkers (*p* 0.05). ROC curve analysis further revealed that area under the curve (AUC) for MIF has a better diagnostic capacity than MCF (AUC 0.981 vs. 0.673).

**Conclusions:**

Our results suggest that MIF could be considered as a potential salivary biomarker for gingivitis.

## Introduction

Current developments in science, especially in biochemistry have a significant impact on the prevention, identification, and management of oral diseases. Oral fluids such as saliva and gingival crevicular fluid contain biomarkers that might suggest changes taking place in the oral cavity and elsewhere in the body [1].

Dental plaque biofilm-induced gingivitis is "an inflammatory lesion resulting from interactions between the dental plaque biofilm and the host's immune-inflammatory response, which remains contained within the gingiva and does not extend to the periodontal attachment (cementum, periodontal ligament and alveolar bone)"[2]. Poor oral hygiene practices of individuals are mainly blamed for the development of gingivitis. In addition, fixed prosthetic restorations with poorly fitted and subgingivally placed margins contribute enhanced accumulation of dental biofilm and eventually gingivitis [3].

Currently, clinical periodontal examination, including the recordings of bleeding from gingiva, edema, color change etc. is the standard to diagnose gingivitis. However, most of the individuals suffering from gingivitis do not seek treatment due to absence of pain in this pathological condition. In addition, these clinical examinations cannot identify the persons at risk of developing periodontitis [4]. Early detection of these undiagnosed cases and monitoring the progressing periodontal disease can be easily achieved by the identification of key salivary biomarkers in gingivitis [5]. Besides, some key evidence has highlighted the moderating effects of micro RNAs (miRNAs) in periodontal tissue homeostasis and they have been linked to the release of proinflammatory cytokines in gingival fibroblasts throughout the initial stages of periodontitis, proposing the crucial modulatory role that miRNAs might have during the initiation and progression of the disease [6].

Generally, plaque induced gingivitis can be treated with meticulous oral hygiene measures and with the adjunctive use of antibacterial and anti-inflammatory mouthwashes [7]. Gingivitis may progress to periodontitis in susceptible individuals if left untreated and this is a common evolution in a large portion of the population [8]. Periodontal pathogens are widely recognized to be responsible for the onset and deterioration of the periodontal conditions, which triggers the host immune response and inflammation to protect against these pathogens [9–11]. In addition, risk factors for the progression to periodontal disease are poor oral hygiene, aging, male gender, diabetes, low socioeconomic status, education level, consumption of alcohol and tobacco [12].

A bacterial invasion at the site of the inflammation activates macrophages, which play a major role in the host's defensive reactions [13–16]. In the initial stage of infection, microorganisms and their products activate the migration of macrophages from the bloodstream to the infection site. Activated macrophages phagocytose the microbes and are in charge of inducing the release of several inflammatory mediators such as tumor necrosis factor-alpha (TNF-alpha), prostaglandin E2, interleukin (IL)-1, and IL-6. These cytokines further induce the development of osteoclasts, which further leads to breakdown of alveolar bone, and the release of proteases such as collagenases and matrix metalloproteinases [MMPs], that alters the periodontal system [17]. Animal and human clinical studies showed the beneficial effects of anti-inflammatory medications such as Tacrolimus that subdued the expression of serum IL-1, TNF-alpha, IL-6 and prostaglandin E2 and protected against the inflammation-induced tissue and bone loss associated with oral lichen planus and periodontitis [18, 19]. Several studies have shown that macrophages’ count in the periodontium increase in periodontitis state in comparison to periodontal health status. Furthermore, macrophages constitute about 5–30% of infiltrating inflammatory cells found in periodontal tissues of periodontitis patients [16, 20]. Chemokines, have a major role in attracting inflammatory cells to the infection site [21]. The essential mediators concerning macrophage activation, accumulation, and function are macrophage activating factor (MAF), macrophage chemotactic factor (MCF), and macrophage migration inhibitory factor (MIF) [22, 23]. These mediators are secreted by monocytes, dendritic cells, neutrophils, eosinophils, mast cells, basophils, endothelial cells, T-lymphocytes and macrophages after stimulation from pathogens and inflammatory cytokines [24]. The role of MCF is to attract macrophages to the infected tissue [25]. MAF activates macrophage phagocytosis action and the ingestion of pathogens [26]. MIF restrains the movement of local macrophages out of local tissue [27]. Number of circulating MIF was found amplified during inflammation [28]. Micro RNA 451a (miR-451a) can precisely target MIF and downregulate its expression and can lead to decreased cell proliferation, colony formation, cell migration [29].

Saliva contains 99% of water combined with electrolytes, immunoglobulins, albumin, enzymes, glycoproteins, and antimicrobial factors [30–32]. Besides, it is considered as one of the critical sources of non-invasive investigations of biomarkers related to periodontal disease [33, 34]. Many inflammatory mediators, including the inflammatory cytokines interleukin-1 beta (IL-1β), tumor necrosis factor-alpha (TNF- α), and prostaglandin E2 (PGE2) have been found in saliva during the development and progression of periodontal disease. These inflammatory mediators are critical in developing periodontal disease [35, 36]. However, very few studies were done to investigate MAF, MIF, and MCF biomarkers in saliva for early diagnosis of gingivitis and progression of periodontal disease [27, 37, 38].

Rational of our study was to explore the levels of these chemokines in gingivitis patients as early biomarkers predicting the progression into periodontitis, since gingivitis progress into periodontitis only in susceptible individuals [8]. Hence, the aim of this study is to compare the salivary levels of MAF, MCF, and MIF in periodontally healthy and gingivitis patients, and correlate levels of these chemo attractants with clinical gingival inflammation levels.

## Materials and methods

### Participants

This study was done at the University Dental Hospital Sharjah, United Arab Emirates from February 2020 to June 2021. Research Ethics Committee from University of Sharjah, approved the study (REC-20-03-10-01-S). Sixty participants, 30 healthy individuals, and 30 gingivitis patients, age range between 18 and 45 years, were enrolled in this research. A verbal and written information were provided to the participants before study enrollment and informed consent was obtained from them.

Inclusion criteria were: participants who were in good general health, age range between 18 and 45 years and have at least 20 teeth present.

Exclusion criteria include: Individuals who have systemic diseases, smokers, alcohol users, pregnant or lactating females, female participants during the menstrual time, patients wearing removable prosthesis or orthodontic appliances, and presence of acute illness (fever, body aches, and diarrhea) at the time of examinations.

### Clinical evaluation

All clinical examinations were performed by the same examiner.

#### Bleeding on probing (BOP)

The BOP was detected by inserting a standardized (dimensions and shape) periodontal probe (UNC 15) to the base of the pocket from six sites (mesiobuccal, buccal, distobuccal, mesiolingual, lingual, distolingual) of all teeth then the presence of bleeding on the site was observed and the percentage of BOP positive units were calculated. The participants who had BOP at ≥ 10% of sites (six sites per tooth) and probing depth (PD) ≤ 3 mm at all sites were considered as having gingivitis. They were further divided into localized gingivitis (BOP in 10–30% of sites) and generalized gingivitis (BOP > 30% of sites) groups. Participants who had BOP at < 10% of sites and PD ≤ 3 mm at all sites were considered as having healthy periodontium [2].

#### Simplified Modified Gingival Index (SMGI)

The level of gingival inflammation was also detected by simplifying Modified Gingival Index (SMGI). The most inflamed area of gingiva was selected and each patient was given a score from 0 to 4. 0: the absence of inflammation, 1: localized mild inflammation (slight change in color, little change in texture), 2: generalized mild inflammation, 3: moderate inflammation (moderate glazing, redness, edema, and hypertrophy and 4: severe inflammation (marked redness and edema/hypertrophy, spontaneous bleeding, or ulceration) [39].

#### Visible Plaque Index (VPI)

The plaque index was utilized to record the amount and the presence of plaque by using a disclosing solution that shows the accumulation of plaque on six surfaces (mesiobuccal, buccal, distobuccal, mesiolingual, lingual, distolingual) of all teeth. The percentage of surfaces with plaque was calculated [40].

### Collection of salivary samples

At the time of the assessment, both groups had a sample of unstimulated saliva taken. Before collecting saliva, participants rinsed with tap water (10 mL) for 30 s and expectorated. Oral hygiene procedures (such as flossing, brushing, and mouth rinses), as well as drinking, eating, and chewing gum, must be avoided for 1 h prior to saliva collection. The amount of unstimulated saliva samples was at least 5 mL and collected in sterile tubes. The samples were kept in an icebox and transferred to be frozen at − 80 °C until analysis [41].

### Biomarker analyses

All the three ELISA kits utilized in the present study were purchased from My Biosource, USA. The salivary levels of Macrophage activating factor (MAF, Cat no: MBS772362), Macrophage inhibitory factor (MIF, Cat no: MBS265761), and Macrophage chemotactic factor (MCF, Cat no: MBS772363) were determined using Enzyme-linked immunosorbent assay (ELISA) kits following the manufacturer's instructions.

### Statistical analysis

Statistical analysis was performed using IBM SPSS statistics for Windows (Computer software) IBM Corp. (version 28). The sample size was calculated based on the results of previous research. A chi-square test was conducted to compare participants’ characteristics among the healthy and gingivitis cases. An independent-sample t-test was conducted to determine the differences in the level of the three biomarkers among healthy and participants with gingivitis. The mean scores of each biomarker were compared among different groups using t test, one-way ANOVA and Kruskal–Wallis tests. The simple modified gingival index groups were analyzed using a one-way ANOVA for the normally distributed MCF biomarker, and Kruskal–Wallis tests were used for the MAF and MIF scores, which were not normally distributed. The effectiveness of biomarkers and prediction panels was further assessed using ROC curve analysis and the accompanying area under the curve (AUC) analysis. The ROC curves were used to determine cut-off values. The cut-off point of the anticipated probability that produced the highest sum of sensitivity and specificity was used to evaluate the sensitivity and specificity for the biomarker combinations. The cutoff for statistical significance was *p* < 0.05.

The effectiveness of biomarkers and prediction panels was assessed using ROC curve analysis and the accompanying area under the curve (AUC) analysis. Using ROC curves, cut-off values were discovered. The cut-off point of the anticipated probability that produced the highest sum of sensitivity and specificity was used to evaluate the sensitivity and specificity for the biomarkers. The cutoff for statistical significance was *p* < 0.05.

## Results

The majority of the study population were females (80.00%) with a mean age of 27.53 ± 6.64. 61.67% of the subjects have university degrees. Regarding their oral health status, half of the sample were healthy (n = 30), and the other half (n = 30) have been diagnosed with gingivitis. The mean of BOP among participants was 14.70 ± 11.86 and the mean of VPI was 55.18 ± 21.26. In terms of oral hygiene, most of the participants (88.33%) brushed their teeth twice during the day and almost half of the subject (53.33%) sometimes used an interdental aid (Table 1).

**Table 1:** Sociodemographic characteristics and clinical parameters of study participants (n = 60)

Category	Frequency	Percent %
*Gender*		
Male	12.00	20.00
Female	48.00	80.00
*Age*		
Mean ± SD	27.53 ± 6.64	
*Level of education*		
Intermediate	23.00	38.33
University	37.00	61.67
*Tooth brushing*		
Twice	53.00	88.33
Once	7.00	11.67
*Interdental aid*		
Daily	18.00	30.00
Sometimes	32.00	53.33
Never	10.00	16.67
*Gingivitis*		
Healthy	30.00	50.00
Gingivitis	30.00	50.00
*Modified Gingival Index*		
Absence of inflammation	8.00	13.33
Localized mild	34.00	56.67
Generalized mild	11.00	18.33
Moderate	7.00	11.67
*BOP* (%)		
Mean ± SD	14.70 ± 11.86	
*VPI* (%)		
Mean ± SD	55.18 ± 21.26	

The results illustrated a non-significant association among the participants’ characteristics except for the VPI values (Table 2).

**Table 2:** Characteristics of study participants among healthy and gingivitis groups

Participants N = 60		Healthy (n = 30)		Gingivitis (n = 30)		*p *value
		N	%	n	%	
Gender	Male	3	25.0	9	75.0	0.053
	Female	27	56.3.0	21	43.8	
Age	< 25	15	50.0	17	56.7	0.482
	25–35	10	33.3	11	36.7	
	> 35	5	16.7	2	6.7	
Age, mean (SD)		28.30 ± 7.07		26.77 ± 6.21		0.278
Education level	Intermediate	8	34.8	15	65.2	0.063
	University	22	59.5	15	40.5	
Tooth brushing	Twice	27	50.9	26	49.1	0.688
	Once	3	42.9	4	57.1	
Interdental aid	Daily	11	61.1	7	38.9	0.106
	Sometimes	12	37.5	20	62.5	
	Never	7	70.0	3	30.0	
VPI		49.70 ± 18.68		60.66 ± 22.54		0.045*

*p < 0.05

**Fig. 1:**
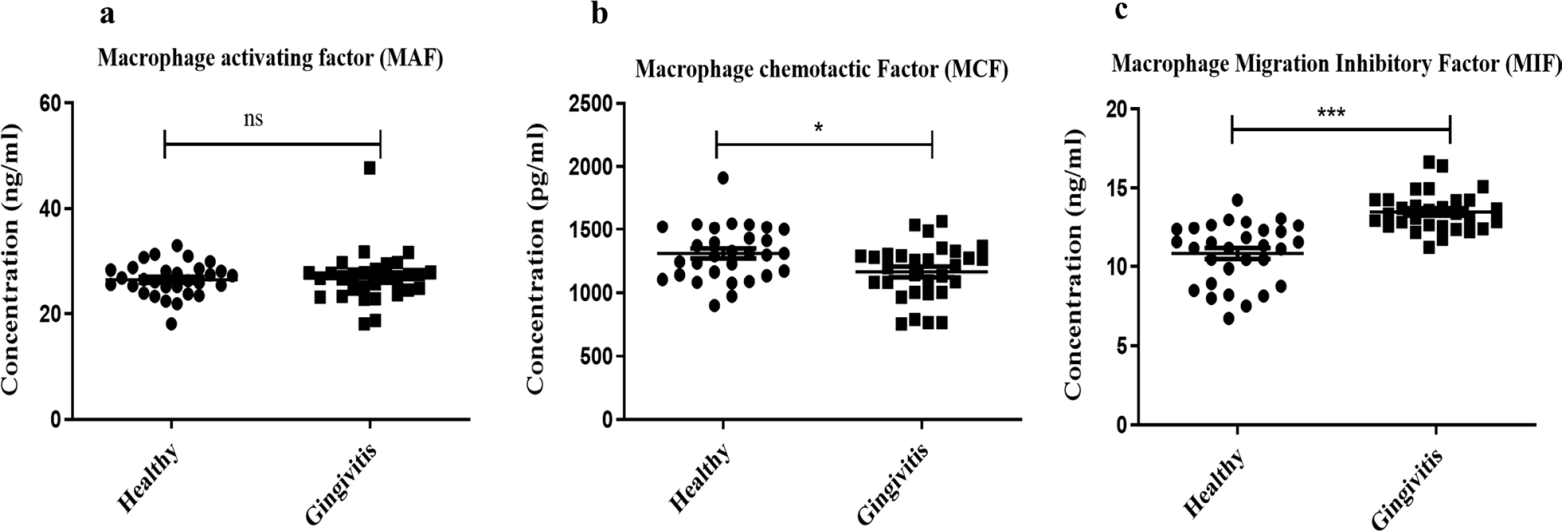
Salivary levels of **a** macrophage activating factor (MAF), **b** macrophage chemotactic factor (MCF) and **c** macrophage migration inhibitory factor (MIF) among healthy and gingivitis cases. *ns* not significant, *p < 0.05 and ***p < 0.001

Among the three biomarkers studied, salivary levels of MAF were not observed to significantly different among the healthy and gingivitis subjects (Fig. 1a). The salivary levels of MCF were however, lower in gingivitis (1164.56 ± 218.08) compared to the healthy subjects (1309.12 ± 213.10), a statistically significant difference of 144.56 was found with *p* < 0.05 (Fig. 1b). On the contrary, the MIF level was higher (13.50 ± 1.26) among gingivitis cases as compared to healthy subjects (10.83 ± 1.93), a statistically significant difference of 2.67 was detected with *p* < 0.001 (Fig. 1c).

Across the simplified modified gingival index groups (absence of inflammation, localized mild, generalized mild, and moderate) no statistically significant difference was found for MCF (*p* = 0.724) and MAF (*p* = 0.118). Whereas, a significant difference in MIF levels was found among the study groups (*p* < 0.05; Table 3).

**Table 3:** Salivary levels of macrophage chemotactic factor (MCF), macrophage activating factor (MAF) and macrophage migration inhibitory factor (MIF) in relation to the simple modified gingival index (SMGI)

	Absence of inflammation	Localized mild	Generalized mild	Moderate	*p* value
		Mean (SD)			
MCF(pg/ml)	1209.30 ± 180.08	1256.48 ± 241.28	1249.23 ± 234.47	1153.45 ± 200.86	0.724
		Median (IQR)			
MAF(ng/ml)	122.80 (20.94)	132.72 (19.69)	133.66 (15.94)	147.10 (24.38)	0.118
MIF	11.55 (2.94)	12.33 (2.98)	13.41 (0.91)	12.55 (1.93)	0.031

**Fig. 2:**
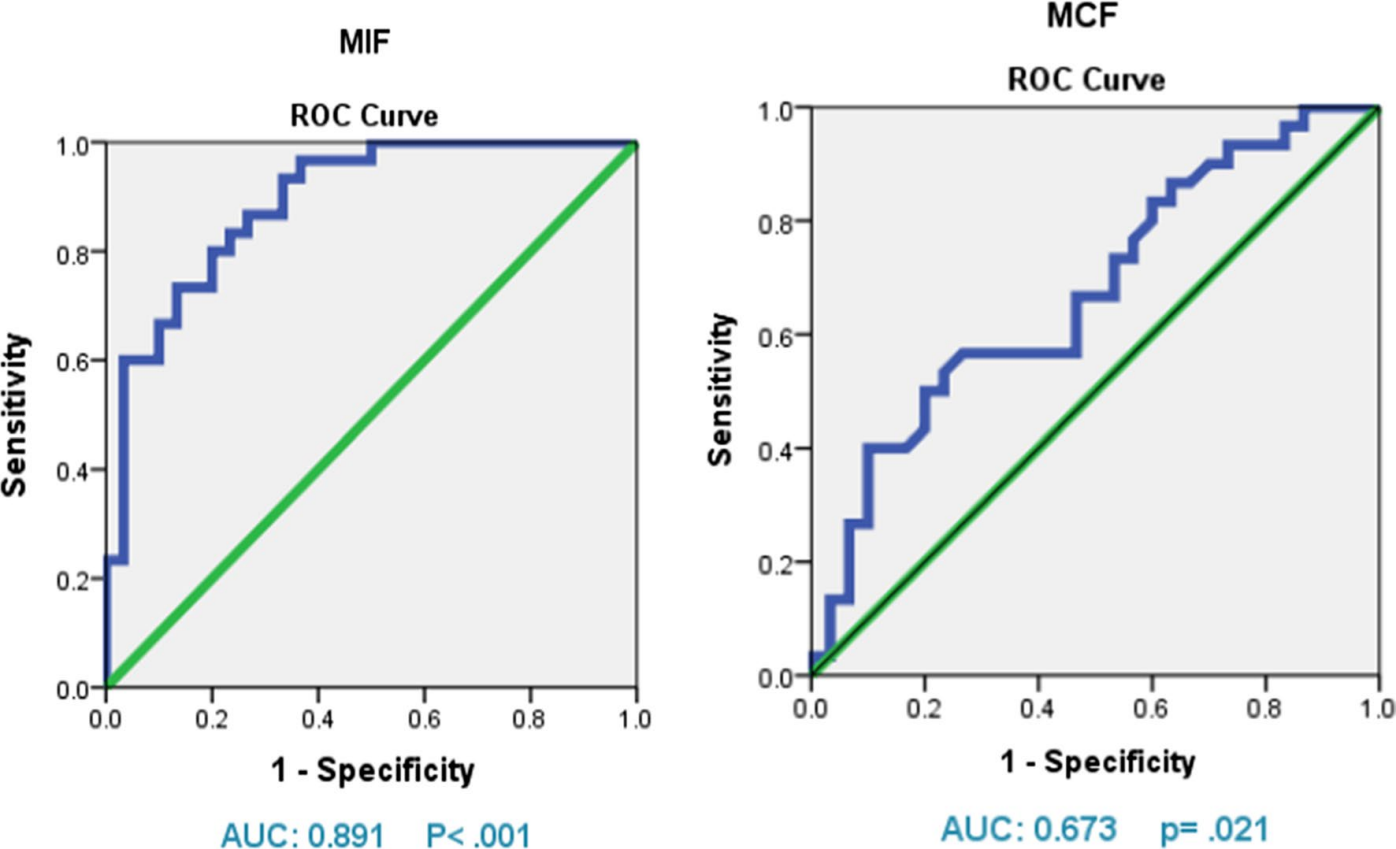
Receiver operating characteristic (ROC) curve test to assess the diagnostic ability of biomarkers (MCF and MIF) in identifying gingivitis cases

Regarding the accuracy of the two tests, the results of the ROC curve and AUC analysis demonstrated that the MIF had a greater capacity for diagnosis than the MCF (Fig. 2). MIF's AUC was 0.981, while MCF's is 0.673. The cutoff point of 12.5, has a sensitivity and specificity value of 0.800. The results from the present study suggested that MIF scores above 12.5 could be plausibly considered to be indicative of gingivitis.

## Discussion

The results of our study showed that among the chemokines associated to macrophage functions, salivary levels of MIF were significantly higher in participants with gingivitis than the ones with healthy gingiva. Besides, we found a significant positive relation between MIF biomarker and clinical gingival inflammation levels. However healthy and gingivitis subjects in our investigation did not differ in their salivary levels of MAF. Even though salivary MCF levels showed significant difference between the groups, it did not reach to the cutoff point to be considered as diagnostic for gingivitis.

Clinicians have been using parameters such as BOP, plaque scores, clinical attachment loss, probing pocket depth, detection of alveolar bone loss in radiographs to diagnose and examine the progression of periodontal disease. However, these methods are time consuming, expensive and their accuracy depends on the skills of clinician [42]. On the other hand, various studies have shown that GCF and saliva could be reliable means to identify the presence and monitor the progression of oral diseases [43, 44]. Even though studies have presented significantly different values of various biomarkers in these oral fluids in periodontal health and disease, so far no biomarker has been identified as having definitive role in diagnosing gingivitis and periodontitis [45]. In the search of these diagnostic biomarkers, we have come across only few studies in the literature regarding those related to macrophage functions [27, 37, 46]. Macrophages have a critical role in defending against periodontal pathogens and periodontal tissue destruction [16]. MCF, MAF, and MIF are significant mediators of macrophage functions [22, 23]. This research analyzed the relationship between salivary MAF, MCF, and MIF levels and clinical gingival inflammation. In order to identify a chemokine associated with macrophage activity for case prediction before case progress into periodontitis, our study examined periodontally healthy and gingivitis patients.

As the periodontal disease progressed through gingivitis and mild, moderate, and severe periodontitis, a previous study found a significant increase in MAF levels [37]. However, healthy and gingivitis subjects in our investigation did not substantially differ in their salivary MAF levels even though we found increased levels of MAF with the increased clinical inflammation, these findings were not statistically significant.

Regarding the salivary MCF levels between healthy periodontium and gingivitis groups, healthy participants showed an increase compared to gingivitis cases. Zhang et al. reported no significant difference of MCF levels between healthy and periodontitis cases, indicating that MCF may not be the primary attractant for macrophage migration to the inflammatory site [37].

We found that salivary MIF levels were higher in gingivitis subjects than the participants with healthy gingiva and the difference in mean MIF levels between healthy and localized gingivitis and healthy and generalized gingivitis group was statistically significant (*p* < 0.0001) (Fig. 1c). Another study noticed that gingivitis patients with and without metabolic disorders had equivalent Gingival Crevicular Fluid (GCF) MIF levels, and similar to our results MIF levels were higher in the gingivitis group compared to the healthy group [47]. In an experimental gingivitis study, Nonnenmacher et al. observed that MIF increased in GCF following 2 weeks of experimental phase compared to baseline levels in young adults. However, they did not find any significant correlation of this result with clinical parameters [38]. Even though study design was different than ours (experimental gingivitis vs naturally occurring gingivitis and biomarkers in GCF vs in saliva), we had similar results. In contrary, Lira-Junior et al. aimed to evaluate levels of biomarkers of innate immunity in serum and saliva of periodontally healthy, gingivitis and aggressive periodontitis patients, no significant differences were found in salivary and serum levels of MIF among the groups [48]. A study conducted by Ortiz-Garcia et al. found that MIF in saliva was greater in chronic periodontitis participants compared to healthy periodontium participants and deduced a correlation between the disease's clinical signs and MIF salivary levels. Thus, MIF biomarker might have an essential function in the pathology and development of chronic periodontitis [27]. Grande et al. reported no significant changes in MIF levels at week 12 after performing non-surgical periodontal treatment. However, there was a considerable reduction in MIF levels from baseline to week 2, suggesting that salivary chemokine concentrations remained constant at the baseline level,throughout the first 2 to 6 weeks following periodontal treatment [49]. As far as we know, MIF promotes leukocyte migration and enrolment to inflammation and infection sites. It responds quickly and strongly to any stimuli, such as microorganisms or their products [50], in addition MIF restrains the movement of local macrophages out of local tissue [27].

Moreover, when we correlated SMGI (clinical gingival inflammation level) to the MAF, MCF, and MIF level, we found a significant positive relation between MIF biomarker and SMGI. In addition, MIF produced higher AUC (0.891) value to discriminate gingivitis patients from healthy subjects, when compared to others.

In conclusion, when we contemplate these findings, we can consider MIF as a potential early diagnostic biomarker for gingivitis and progression to periodontitis among the tested three chemokines namely MAF, MCF and MIF. Further studies are required to investigate the salivary and gingival crevicular fluid levels of these mediators of macrophage functions and micro RNAs targeting/ regulating their synthesis in patients suffering from gingivitis and various stages and grades of periodontitis and in response to nonsurgical and surgical periodontal therapy.

## Data Availability

The data produced during this research is available from the corresponding author on reasonable request.

## Abbreviations

MAF: Macrophage activating factor
MCF: Macrophage chemotactic factor
MIF: Macrophage migration inhibitory factor
SMGI: Simplified Modified Gingival Index
BOP: Bleeding on probing
VPI: Visible Plaque Index
GCF: Gingival crevicular fluid
ROC: Receiver operating characteristics
AUC: Area under the curve
